# The Metabolic Role of Ketogenic Diets in Treating Epilepsy

**DOI:** 10.3390/nu14235074

**Published:** 2022-11-29

**Authors:** Kaleem Imdad, Turki Abualait, Ammara Kanwal, Ziyad Tareq AlGhannam, Shahab Bashir, Anum Farrukh, Sahir Hameed Khattak, Raidah Albaradie, Shahid Bashir

**Affiliations:** 1 Department of Biosciences, COMSATS University Islamabad, Islamabad 45550, Pakistan; 2 College of Applied Medical Sciences, Imam Abdulrahman Bin Faisal University, Dammam 34212, Saudi Arabia; 3 College of Medicine, Imam Abdulrahman Bin Faisal University, Dammam 34212, Saudi Arabia; 4 Department of General Medicine, Fauji Foundation Hospital, Rawalpindi 45000, Pakistan; 5 National Institute for Genomics and Advanced Biotechnology (N.I.G.A.B.), National Agriculture Research Centre (NARC), Islamabad 44000, Pakistan; 6 Neuroscience Center, King Fahad Specialist Hospital Dammam, Dammam 32253, Saudi Arabia

**Keywords:** epilepsy, drug-resistant epilepsy, biomarkers, parameters, ketogenic diet

## Abstract

Epilepsy is a long-term neurological condition that results in recurrent seizures. Approximately 30% of patients with epilepsy have drug-resistant epilepsy (DRE). The ketogenic diet (KD) is considered an effective alternative treatment for epileptic patients. The aim of this study was to identify the metabolic role of the KD in epilepsy. Ketone bodies induce chemical messengers and alterations in neuronal metabolic activities to regulate neuroprotective mechanisms towards oxidative damage to decrease seizure rate. Here, we discuss the role of KD on epilepsy and related metabolic disorders, focusing on its mechanism of action, favorable effects, and limitations. We describe the significant role of the KD in managing epilepsy disorders.

## 1. Introduction

Epilepsy is one of the most common neurological disorders worldwide, with a prevalence of 0.5–1% and a lifetime incidence of 1–3% [1]. Individuals with epilepsy have uncontrollable seizures due to irregularities in brain activity [2]. The variance between excitation and inhibition in neural circuits plays a vital role in epilepsy’s pathogenesis [3]. There is a long-term propensity to suffer from epileptic seizures and the associated neurological, cognitive, and psychosocial implications of epilepsy [2].

Approximately 30% of people with epilepsy will continue to have seizures even when taking multiple anti-epileptic drugs (AEDs) [4]. Approximately 20–35% of children with epilepsy are drug-resistant [5]. Epilepsy is considered drug-resistant when two trials of adequate, tolerated, appropriately chosen and scheduled AEDs fail to achieve seizure control [5]. Uncontrolled seizures pose a significant risk to epilepsy patients’ quality of life [6]. The ketogenic-diet (KD) is a high-fat, adequate-protein, and low-carbohydrate diet. Due to the emerging evidence around the effectiveness of KD in managing drug-resistance epilepsy (DRE), the interest in studies to find the metabolic alterations that could potentially control epilepsy has increased [7].

In addition, the KD is garnering increasing consideration as a possible therapy for various neurological illnesses [8]. Recent research on newborns with epilepsy has demonstrated that KD is extremely effective and well-tolerated [9]. We performed a literature review taking into account all relevant published studies available online (Internet, PubMed, etc.), including case reports, case series, conference abstracts, and retrospective and prospective studies that evaluate the metabolic role of KD for treating epilepsy from 2009 to 2022. This review summarizes the evidence supporting the anti-seizure and neuroprotective properties of KD in epilepsy patients.

## 2. Research Methodology

All studies were investigated by using selected key words on various databases for instance, PubMed, PMC, Google Scholar, Science Direct, and Oxford Academia. 

Only those studies were included which had primarily focused on efficacy of ketogenic diet, reduction in epileptic seizures or both. The studies must illustrate the seizure reduction percentage. The parameters used are references, year of publication, total number of people screened with epilepsy, type of treatment given to epileptic patients, gender and age distribution, time duration of treatment, name of area, type of epilepsy and seizures reduction percentage among patients, and study design employed in selected date.

## 3. Study Results

### 3.1. Epilepsy 

Epilepsy is bimodally distributed with two peaks at both extremes of life: it is highest in the first year, then incidence drops to adult levels by the age of 10, before incidence rises again in people over the age of 85 years [10]. Incidence is higher in low-income countries, and usually above 80–100 per 100,000 persons per year for unknown reasons, but sub-standard health-delivery system, poor hygiene, lack of basic sanitation, and a higher risk of infections and traumatic brain injury may contribute [11,12].

**Figure 1:**
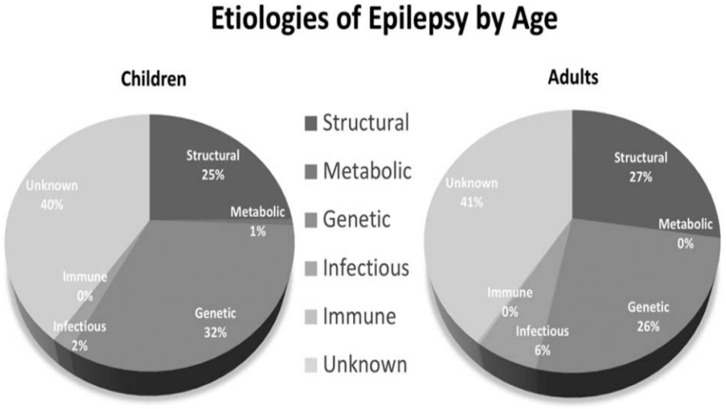
Etiologies of epilepsy by age.

Epilepsy is defined as: (1) two unprovoked seizures occurring more than 24 h 24 apart; or (2) a single unprovoked seizure if recurrence risk is high (i.e., >60% over the next 10 years) or (3) a diagnosis of an epilepsy syndrome [12]. Figure 1 shows the etiologies of epilepsy at various ages [13]. 

The process converting a non-epileptic brain into one capable of generating spontaneous, recurrent seizures is known as “Epileptogenesis”. The process is conceptualized to result from an imbalance between excitatory and inhibitory activity within a neuronal network, becomes more disposed to fire in an excessive, hypersynchronous, oscillatory manner which when sustained, disrupts normal neuronal processing, and is capable of recruiting other neuronal networks [10,11].

During the past several decades, neuroimaging, genomics, and molecular biology have substantially improved our knowledge of the pathophysiology of seizures and epilepsy [14,15]. Seizure is the main incident indicator found in epilepsy that is related with high persistence pulse, emitted from a set of neurons [16]. Seizures can present in various forms; seizures can present with motor symptoms or behavioral changes. Seizures can also happen with the patient aware or unaware [17].

### 3.2. Prevalence 

Epilepsy affects 1–2% of people worldwide [18,19,20]. It is estimated that 23 million Asians suffer from epilepsy, while only 3.3 million Africans are affected by this disease [10]. Epilepsy affects both males and females of all ages. Focal seizures are common in children and adults [10].

### 3.3. Diagnosis

After carful history taking and examination, techniques such as neuroimaging, neurophysiological studies, and lab tests are used in diagnosing epileptic seizures and related disorders [21]. 

### 3.4. Mortality

Epilepsy death rates are relatively high in the USA and UK [22]. One study has linked epilepsy to 15% of deaths, and it remains unclear how to lessen this risk, while other studies reported 87.5% mortalities due to non-epileptic reasons [23]. Age, generalized seizures, and other independent variables raise the risk of death among epileptic patients [23,24]. 

### 3.5. Epilepsy Therapy 

Epilepsy patients must be managed in the aim to become seizure-free. The type of epileptic syndrome determines treatment, as does the patient’s age, gender, and acceptance [25]. 

### 3.6. Medicines

AEDs work by boosting neurotransmitters or decreasing excitatory processes [26]. In the US, phenytoin is considered a frequently used AED. Unfortunately, its metabolic role in the liver and random pharmacokinetics is not fruitful for older individuals [26]. 

Although there are several therapies, the treatment of epilepsy is based mainly on drugs, which, depending on the year of coming onto the market are classified as first, second, or third generation. The new-generation (third generation) AEDs may offer better tolerability, milder adverse effects, less drug interactions and improved pharmacokinetic characteristics compared to the conventional AEDs. For this reason, the New-generation AEDs may be used earlier in epileptic patients. Further head-to-head comparisons are needed to determine the exact position of New-generation AEDs relative to conventional AEDs, because, despite advancements and the development of New-generation AEDs, a third of patients with epilepsy remain refractory to pharmacotherapy [27].

Nanomaterials or nanomedicine, especially biosensor-based methods, can facilitate the analysis of these agents with unique advantages such as rapid analysis, sensitivity, selectivity, and low cost. Additionally, various chemical and biological modifiers to improve the sensitivity and selectivity of the sensor have been also been categorized [28]. These new molecules have been developed in order to provide a pharmaceutical profile and tolerance superior to the previously available drugs, and it is forecast that as their use increases, their true potential and profile will be widen. Furthermore, for the first time in Paediatric Epileptology, the extrapolation of the efficacy data in adults have been also been used (together with specific safety and pharmacokinetic studies in the paediatric population), in order to speed up their approval for use in the child population in upcoming years [29]. Table 1 summarizes the some of the used drugs and their role in controlling epilepsy [30]. 

### 3.7. Surgery

Epileptic surgery is a potentially curative treatment for children with refractory seizures. Early epileptic surgery has been emphasized to treat medically intractable epilepsy in children. Seizure reduction results in remarkable developmental and cognitive improvements. Prolonged invasive extraoperative electroencephalography (EEG) or stereoEEG monitoring with depth electrodes and/or subdural grids are usually used for patients with nonlesional MRI or discordant EEG epileptogenic zones [31]. Epileptic surgery is among the most successful methods to achieve a seizure-free status [32,33]. Approximately 50%–80% of patients became seizure-free after surgery [34]. 

### 3.8. Dietary Treatment

In drug-resistant epilepsy, diet alteration is an alternative non-pharmacological option to treat epileptic seizures and is widely used to treat glucose transporter type 1 deficiency syndrome (GLUT1 DS), pyruvate dehydrogenase deficiency [35]. The KD has long been used to treat epileptic seizures [36]. The potential of the KD to control epileptic seizures has been known about for a century in medical and research institutes. Additionally, efforts are being made to recognize the KD’s therapeutic role in treating acute and severe metabolic disorders [37].

### 3.9. Ketogenic Diet

KD is defined as a diet containing a high amount of fat, low in carbohydrates, and with adequate protein content. It was first designed in the 1920s to treat seizures and supplies energy through ketone bodies (KBs) to the brain when the glucose level is lower in the body [38]. There are three main KBs: β-hydroxybutyrate (BHB), acetoacetate (ACA), and acetone. KBs act as fuel elements and are mainly formed from fatty acids by the liver during starvation and exercise [39]. The medium-chain triglycerides diet (MCTD) consists of high fat content with low glycemic index (LGI) [40]. Ketogenesis is a metabolic process that provides the body with an alternative form of energy through the production of KBs [41]. In ketogenesis, acetyl-CoA derived from β-oxidation of fatty acids is converted into KBs in the mitochondrial matrix of liver cells and then these ketone bodies are carried to the extrahepatic tissues for alternative energy sources. Adenosine has long been linked to metabolic and neural activity, and studies have proven that a ketogenic diet suppresses seizures by increasing inhibitory effects mediated through adenosine A_1_ receptors [8,42]. 

The ketone bodies, which are derived from fatty acid oxidation and usually produced in fasting state or on high-fat diets, have broad neuroprotective effects [43]. It is also suggested that the insulin sensitivity increased during a Ketogenic meal [44]. Furthermore, the neuroprotection and homeostasis also promotes the activation of inhibitory adenosine A1 receptors (A1Rs) by dephosphorylating extracellular ATP to adenosine [45]. Also, it activates GIRKs, which are G protein-coupled inwardly rectifying K+ channels. KATP channels activation may also be linked to A1R activation by a KD [46]. Another molecular relationship exists between the KD and γ-aminobutyric acid (GABA) levels and KATP channel activation through GABAB receptors. KATP channels activation has also been reported by other stimulants such as xanthine, diazoxide, etc. KATP channels play basic roles in nerve, muscle, epithelial, and endocrine tissue physiology and their direct activation regulates pancreatic islet β-cell membrane potential, calcium influx, and insulin secretion, and rectifies drug targets for metabolic disorders of glucose homeostasis [47]. Enhanced PIP_3_ signaling in pro-opiomelanocortin (POMC) neurons causes a K_ATP_ channel activation that leads to diet-sensitive obesity. In a mice study, a POMC neurons showed a marked hyperpolarization and a reduction in basal firing rate due to increased ATP-sensitive potassium (K_ATP_) channel activity as well. The K_ATP_ blocker (e.g., tolbutamide) restored electrical activity and leptin-evoked firing of POMC neurons in mice. These data indicate that PIP_3_-mediated signals are critical regulators of the melanocortin system via modulation of K_ATP_ channels [48]. In another study it was well documented that K_ATP_ channel blockers control glucagon secretion by distinct mechanisms i.e., a direct stimulation of α-cells involving a [Ca^2+^]_c_ rise and an indirect inhibition mediated by somatostatin. By closing α-cell K_ATP_ channels, sulfonylureas depolarize α-cells, increase [Ca^2+^]_c_, and stimulate glucagon secretion. However, their effects also involve an indirect inhibitory effect via somatostatin (SST) secreted by δ-cells on the glucose concentration [49,50].

Reactive oxygen species (ROS) may be reduced by metabolic modifications, improving seizure resistance (ROS). Fructose 1,6-bisphosphate administered to rats shifts glucose consumption to the pentose phosphate pathway [51].

### 3.10. Types of KD

KD is widely used to treat patients with refractory epilepsy or those individuals unfit for surgical management [52]. There are four types of KD [53].

Classic KD: In the classic KD, the ratio of fat and carbohydrates is 4:1. This ratio can be altered to 3:1 for moderate metabolism activity [7].

Medium Chain Triglyceride (MCTD): This modified Atkin diet includes high production of KBs than any other class of fats, such as long-chain triglycerides (LCT) [1]. It can lower the intake of fatty acids due to its ketogenic properties and greater carbohydrate and protein content due to its ketogenic properties because it contains high fat content (60%) and lower carbohydrate and protein ratio. Moreover, it also leads to marked alterations in brain energy metabolism, with ketone bodies partly replacing glucose as fuel. Though the phenomena is still not completely understood, it is reported that the ketone body acetone has anticonvulsant activity and could play a role in the seizure protection afforded by the diet [53]. In addition to acute seizure protection, the ketogenic diet provides protection against the development of spontaneous recurrent seizures in models of chronic epilepsy, and it has neuroprotective properties in diverse models of neurodegenerative disease [54,55]. The MCTD diet is more flexible for children than other KD because it increases the growth rate, decreases the requirement for other micronutrients, and has a lower cholesterol ratio [53].

Low glycemic index treatment (LGIT): LGIT is a non-restrictive treatment that consists of a diet with an increased amount of fat (60%), a high amount of protein (20 to 30%), and 10% carbohydrates [7]. It comprises foods with a low glycemic index (i.e., mutton, few fruits, dairy food) [55]. The fat:carbohydrate:protein ratio is about 1:6:0. There are no restrictions on diet and calories intake. Although LGIT represent fewer KBs than another KD, it is a better-tolerated diet. 

Modified Atkins Diet (MAD): MAD constitutes 65% fat content, 25% protein, and a low carbohydrate intake (10%). The fat ratio is high in MAD and is considered the most savory form of KD and acceptable for adults or individuals suffering from behavioral issues [56]. There is no specific amount restriction of liquid or protein intake, but the carbohydrates amount is fixed as 10 to 20 g/day in infants and 15 to 20 g/day in youngsters. It is also recommended to take an appropriate calcium supplementation and a KD [57,58] because sufficient vitamins and minerals are normally found in a well-balanced diet. However, due to the limited quantities of fruits, vegetables, enriched grains, and foods containing calcium in the KD, supplementation is essential, especially vitamins B and C. Previous study also suggests that there is little vitamin D and calcium in KD and evidence for decreased Vitamin D levels in children with epilepsy, and therefore both vitamin D and calcium should be supplemented [59].

### 3.11. Biochemistry of KD

At the beginning of KD utilization, blood glucose becomes low and stabilizes, by which insulin release stops, and the body goes into a catabolic condition [53]. If insulin is depleted further through a KD and the gluconeogenesis process does not favor the metabolic reactions, free fatty acids (FFAs) are utilized by the body and provide power to the brain as the primary energy reservoir [39]. The brain can use KBs produced by the oxidation of FFAs in the absence of glucose [7,55,60]. Although the brain demands less glucose when KB levels are between 2 and 4 mM in the blood, these KBs might only meet up to 60% of brain activity demand [41]. 

After entering into mitochondria, fatty acids are separated and converted into acetyl-CoA by the action of β-oxidation. Large amounts of acetyl-CoA are generated when the liver deletes FFAs excessively by applying classic KD [61]. Oversupply of acetyl-CoA initiates the production of KBs through ketogenesis. Less insulin stimulates enzymatic transformation that results in ACA production from two molecules of acetyl-CoA that are further converted into acetone bodies or BHB [62]. These are the three KBs generated in the bloodstream during a prolonged period of KD application. These KBs can either be moved to the brain through monocarboxylate transporter 1 (MCT-1) [36] or can be removed via urination. After entering the brain, KBs are again changed into acetyl-CoA that reaches the tricarboxylic acid (TCA) cycle [60]. One of the significant roles of KBs is that it generates more ATP molecules than glucose, establishing metabolic activity even in starvation or caloric limitation [63].

Ketogenesis is a functional modification to a malnourishment condition or a diet consisting of lower carbohydrate function as KBs present in the bloodstream in the range of 2–5 mM for 1–2 weeks [64]. Ketogenesis never implicates a change in acid-base stability or reducing blood pH. By constantly raising the amount of KBs in the bloodstream and the presence of lipids and blood glucose, the alleged state of beneficial ketosis is established by KD introduction [41]. Ketosis is a naturally occurring process in response to low glucose supply and is involved in the continuity of human life. It acts as an alternative fuel reservoir for brain and muscle tissues, compensating for glucose deficiency [36].

### 3.12. Efficacy of the KD

KD treats patients with epilepsy ranging from infant to adult age. In the beginning, it was rare that KD would be recommended for adults and older candidates, but research has shown that it is equally beneficial for adults [65,66]. However, KD is more effective in some epilepsy-related syndromes than in others. For example, the reduction rate of epileptic seizures is higher in patients suffering from Dravet syndrome or infantile spasms when KD is introduced [67]. 

By following the KD, children with epilepsy who are resistant to other treatments can achieve reduced ghrelin and des-acyl ghrelin levels. If ghrelin availability is reduced for long periods, this might account for the reported growth retardation seen in children on a KD for an extended time [68,69]. Plasma ghrelin levels in children with refractory epilepsy are consistently lowered and sustained on the KD. Low growth indices in most patients were linked to this alteration [68]. Plasma ghrelin levels in children with refractory epilepsy are consistently lowered and sustained on the KD [69]. However, multiple studies have shown that the KD is directly associated with seizure rate in epilepsy patients [70]. KD has been shown to be beneficial in some epilepsy syndromes and ineffective in others as shown in (Table 2) [59].

Variables that could have influenced the interpretation of the data have also been identified, e.g., gut microbiota [52].

### 3.13. Increased Bio-Energetic Reaction

KBs stimulate and ensure a more significant amount of energy for the brain than energy generated by glucose. Researchers have reported that ketones prompt the regulation of genes involved in a metabolic reaction that leads to the enhancement of mitochondria in neurons [71]. It is proposed that the maintenance of neurons at resting potential can be improved by decreasing the rate of seizures, which is possible by actively metabolic functions. Improved ATP production might give power to the cell, so they function correctly and stabilize the function of ion channels and transporters, increasing the energetic rate and thus maintaining the neuronal equilibrium by providing resistance to internal head injuries during seizures attacks. The reduction of glutamate in the brain can be caused by the formation of GABA receptors, which is possible using KD. Moreover, the anti-seizure process can be developed [72].

### 3.14. Anti-Inflammatory and Antioxidative Behavior

KD regulates the antioxidative shield within the body by increasing glutathione supply and guarding mitochondrial DNA from oxidative stress. This antioxidant mechanism prevents neuronal cells from being damaged by epileptic seizures [70]. KD regulates mitochondria’s coupling protein, which inversely reduces ROS production and provides resistance against episodic seizures [36]. KBs are involved in energy generation and constitute multiple functions that work together to reduce the seizures rate. For example, BHB acts as pleiotropic activity toward histone deacetylases and interacts with immune cells [73]. 

However, there is no proof that KD remarkably decreases the pH of the brain. Little pH change may occur due to cellular membrane changes. Numerous receptors are regulated by pH, such as GABA receptors associated with epileptic seizures activity [69,70].

### 3.15. KD and Pathophysiology of Epilepsy

**Figure 2:**
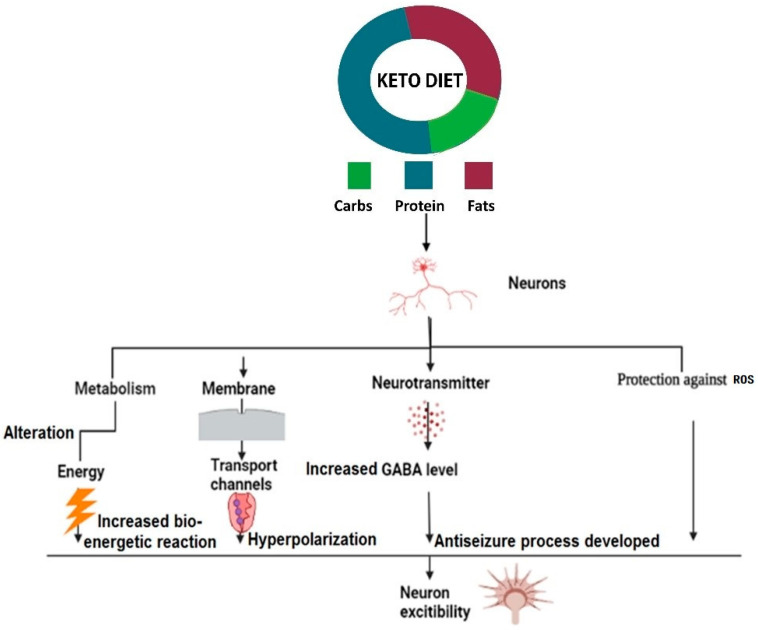
The introduction of ketogenic diets reported an enhancement of chemical messengers in the brain.

How the KD suppresses the reduction rate of seizures in epilepsy patients is not fully known. It has been suggested that KBs and some unsaturated fatty acids play an essential role in generating anti-seizure activity among epilepsy patients [74]. KB induces changes in chemical messengers and alterations in neuronal metabolic activity to regulate neuroprotective mechanisms towards oxidative damage to decrease the rate of seizures [75]. The introduction of KD in epilepsy patients reported an enhancement of chemical messengers in the brain, i.e., GABA, agmatine, monoamines, and reduced neurons irritability, thus building up an anti-seizure state among patients. In the central nervous system (CNS), the production of GABA is increased while the quantity of aspartate inhibitors decreases [74] (Figure 2). A reduced aspartate level due to ketosis is involved in the stimulation of glutamate, which is further changed into glutamine. This glutamine assimilates by neuron cells and causes its conversion into GABA, which acts in an inhibitory manner to reduce oxidative stress [76]. KD also stimulates the overexpression of protein molecules, such as neuropeptides [77]. KBs regulate the potassium channels to ensure the continuous supply of potassium ions. Special fats, which are defined as a type of plastic fat having soft and solid consistency with broad melting range, such as butter, tallow, and lard, etc., are commonly used in the preparation of traditional fast-frozen foods (dumplings, patra, samo, spring rolls) and play a vital role in the desirable textural properties of these food products [78], while their introduction into the diet can help in reducing seizures by suppressing sodium gated channels and calcium channels [79].

### 3.16. Administration of KD

The administration of KD depends on its qualitative and quantitative nutritional value along with time management and should be administered through neurologist. Biochemical test findings and medications related to seizure control must be considered, and other tests to determine disease pathophysiology are often necessary [80]. Most epileptic patients recover after using KD for almost 3 months [81]. Regular checkups are mandatory to assess the nutritional and seizure condition in patients during the application of KD. The use of coconut oil boosts ketosis and decreases the risk of adverse effects from the KD [82]. Children could enjoy sweet and heavy foods, such as butter and cream, for KD [83], while older individuals cannot digest a high proportion of fats due to the slower activity of their digestive system. Therefore, they are initially tested with a moderate KD. 

### 3.17. Acceptability and Adverse Effects of KD

Use of KD in epilepsy type in which individuals are resistant to medicines or drugs shows a satisfactory seizure reduction rate [52]. Some case studies and case series demonstrate that KD can have some antidepressant and mood stabilizing properties, however, no clinical data is available yet [84]. Classic KD gives more rectification than MAD [85]. In that case, it may lead to side effects within the body that decrease the efficacy of the therapeutic nature of KD [86]. Patients’ dropout ratio is entwined with the effectiveness of treatment. Side effects appear at the initial dietary intake like diarrhea, vomiting, acidosis in metabolism, and loss of appetite [87]. As these complications are secondary and known, these can be avoided by a metabolic shift that does not constrict the diet continuation. 

In the young population, the risk of cardiovascular disease may increase as the diet contains a high quantity of fats that generates poor cholesterol levels [87]. Thus, minor adjustments in diet intake can improve the effectiveness of KD therapy, thereby reducing the dropout rate [86].

## 4. Importance of KD

The discovery of new methods and technology has helped humanity in many ways [88,89,90,91], and one of the common examples is the KD, which has been successfully used to manage seizures [92]. The employment of KD for treating neurological disorders, such as epileptic seizures is increasing worldwide. The potential of KD to cure epileptic seizures and the generation of neuronal activity has been seen for a century in medical and research institutes. Additionally, efforts are being made to recognize the therapeutic role of the KD in metabolism to treat acute and severe disorders [37]. It is found that starting three months of KD therapy is referred to as an initial examination, so the dropout period of patients must be conducted in the continuing months of treatment. Individuals following KD therapy (with the fat: carbohydrate ratio of 4:1 in their diet) a reduction in seizures of greater than 50% during 2–3 months of KD therapy [92,93]. 

Together with the changes in indications, the clinical management and administration of the diet have also evolved. Where the early protocols were restrictive with prolonged fasting, today the diet is often started on an outpatient basis without the need for fasting [94]. Moreover, while initially all foods had to be carefully weighed, currently there are KDTs with “free foods”, such as the MAD [95] or formulas [59] and parenteral ketogenic solutions [96]. The much feared adverse effects have proven to be largely preventable [96], although there is still a gap in the knowledge regarding long-term complications, such as growth and cardiovascular alterations [96,97].

Over time, the KD has been considered as first choice in the treatment of epilepsy along with specific metabolic disorders, such as Glucose Transporter Protein 1 (GLUT-1) deficiency syndrome and pyruvate dehydrogenase deficiency [98]. 

Moreover, it is concluded that patients drop out of KD studies because of side effects [57,99]. The KDs is not a common practice all-around and still requires a lot of researches for its recommendation as a primary general treatment [8]. Therefore, there is a need to practice KD treatment in other countries as well. To encourage the wider application of the KD worldwide, the International League Against Epilepsy provided a special description listing the basic and least requirements needed for the employment of KD treatment in resource deficient areas as well as for establishing KD workshops for physicians and nutritionists in South Asian regions [100]. 

## 5. Conclusions

This review provides support for the effectiveness of the KD in treating epileptic seizures in both young and older populations. There is a need for further research with extensive sample data to assess the association of KD with epileptic seizures and syndromes. Various types of epilepsy have been explained in our review, but the efficacy depends not only on the seizure rate, but also on the seizure type. There are limited studies to support the treatment of epilepsy through KD in elderly patients with epileptic seizures. Although there are several therapies, the treatment of epilepsy is based mainly on drugs, which, depending on the year of coming onto the market, are classified as first, second, or third generation The New-generation (third generation) AEDs may offer better tolerability, milder adverse effects, fewer drug interactions, and improved pharmacokinetic characteristics compared to the conventional AEDs. Despite advancements and the development of new-generation antiepileptic drugs (AEDs), some patients remain refractory to pharmacotherapy. So far, KD has proven quite effective, but more research is needed to assess the efficacy of KD therapy. Additionally, more studies and clinical evidence are needed to determine the precise mechanism of KD. 

## Figures and Tables

**Table 1:** Common Drugs and their Molecular Target.

Drugs	Molecular Target
**Voltage-gated ion channels**	
Phenytoin, fosphenytoin, carbamazepine, oxcarbazepine, eslicarbazepine acetate, lamotrigine, lacosamide; possibly topiramate, zonisamide, rufinamide	Voltage-gated sodium channels
Ethosuximide	Voltage-gated calcium channels (T-type)
Ezogabine	Voltage-gated potassium channels (K_v_7)
**GABA inhibition**	
Phenobarbital, primidone, benzodiazepines, including diazepam, lorazepam, and clonazepam; possibly topiramate, felbamate, ezogabine	GABA_A_ receptors
Tiagabine	GAT1 GABA transporter
Vigabatrin	GABA transaminase
Levetiracetam	SV2A
Gabapentin, gabapentin enacarbil, pregabalin	α2δ
**Ionotropic glutamate receptors**	
Perampanel	AMPA receptor
Valproate, felbamate, topiramate, zonisamide, rufinamide, adrenocorticotrophin	Mixed/unknown

**Table 2:** Effectiveness KD in Epilepsy condition.

S.No	KD Beneficial	KD Contraindications
1	Adenylosuccinate lyase deficiency64	Carnitine deficiency (primary)
2	CDKL5 encephalopathy	Carnitine palmitoyltransferase (CPT) I or II deficiency
3	Childhood absence epilepsy	Carnitine translocase deficiency
4	Cortical malformations	β-oxidation defects
5	Epilepsy of infancy with migrating focal seizures	Medium-chain acyl dehydrogenase deficiency (MCAD)
6	Epileptic encephalopathy with continuous spike-and-wave during sleep	Long-chain acyl dehydrogenase deficiency (LCAD)
7	Glycogenosis type V	Short-chain acyl dehydrogenase deficiency (SCAD)
8	Juvenile myoclonic epilepsy	Long-chain 3-hydroxyacyl-CoA deficiency
9	Lafora body disease	Medium-chain 3-hydroxyacyl-CoA deficiency.
10	Landau-Kleffner syndrome	Pyruvate carboxylase deficiency
11	Lennox-Gastaut syndrome	Porphyria
12	Phosphofructokinase deficiency	Inability to maintain adequate nutrition
13	Rett syndrome	Surgical focus identified by neuroimaging and video-EEG monitoring
14	Subacute sclerosing panencephalitis (SSPE)	Parent or caregiver noncompliance

Note: Data has been taken from Kossoff et al., 2018 [64].

## Data Availability

Not applicable.

## References

[1] Verrotti A., Iapadre G., Di Francesco L., Zagaroli L., Farello G. (2020). Diet in the Treatment of Epilepsy: What We Know So Far. Nutrients.

[2] López S.V., Ramos-Jiménez C., de la Cruz Reyes L.A., Ruiz A.K.G., Arriola L.A.B., Olivares J.M.M., Galindo E.G.A., Pedroza I.F.P., San-Juan D. (2021). Epilepsy diagnosis based on one unprovoked seizure and ≥60% risk. A systematic review of the etiologies. Epilepsy Behav..

[3] Yamagata A., Miyazaki Y., Yokoi N., Shigematsu H., Sato Y., Goto-Ito S., Maeda A., Goto T., Sanbo M., Hirabayashi M. (2018). Structural basis of epilepsy-related ligand–receptor complex LGI1–ADAM22. Nat. Commun..

[4] Granata T., Marchi N., Carlton E., Ghosh C., Gonzalez-Martinez J., Alexopoulos A.V., Janigro D. (2009). Management of the patient with medically refractory epilepsy. Expert. Rev. Neurother..

[5] Lyons L., Schoeler N.E., Langan D., Cross J.H. (2020). Use of ketogenic diet therapy in infants with epilepsy: A systematic review and meta-analysis. Epilepsia.

[6] Martin-McGill K.J., Bresnahan R., Levy R.G., Cooper P.N. (2018). Ketogenic diets for drug-resistant epilepsy. Cochrane Database Syst. Rev..

[7] D’Andrea Meira I., Romão T.T., Pires do Prado H.J., Krüger L.T., Pires M.E.P., da Conceição P.O. (2019). Ketogenic Diet and Epilepsy: What We Know So Far. Front. Neurosci..

[8] Ko A., Kwon H.E., Kim H.D. (2022). Updates on the ketogenic diet therapy for pediatric epilepsy. Biomed. J..

[9] Armeno M., Verini A., Caballero E., Cresta A., Valenzuela G.R., Caraballo R. (2021). Long-term effectiveness and adverse effects of ketogenic diet therapy in infants with drug-resistant epilepsy treated at a single center in Argentina. Epilepsy Res..

[10] Beghi E. (2020). The Epidemiology of Epilepsy. Neuroepidemiology.

[11] Thijs R.D., Surges R., O‘Brien T.J., Sander J.W. (2019). Epilepsy in adults. Lancet.

[12] Perucca P., Bahlo M., Berkovic S.F. (2020). The Genetics of Epilepsy. Annu. Rev. Genomics. Hum. Genet..

[13] Falco-Walter J. (2020). Epilepsy-Definition, Classification, Pathophysiology, and Epidemiology. Semin. Neurol..

[14] Pressler R.M., Cilio M.R., Mizrahi E.M., Moshé S.L., Nunes M.L., Plouin P., Vanhatalo S., Yozawitz E., de Vries L.S., Puthenveettil Vinayan K. (2021). The ILAE classification of seizures and the epilepsies: Modification for seizures in the neonate. Position paper by the ILAE Task Force on Neonatal Seizures. Epilepsia.

[15] Scheffer I.E., Berkovic S., Capovilla G., Connolly M.B., French J., Guilhoto L., Hirsch E., Jain S., Mathern G.W., Moshé S.L. (2017). ILAE classification of the epilepsies: Position paper of the ILAE Commission for Classification and Terminology. Epilepsia.

[16] Baumgartner C., Koren J., Britto-Arias M., Schmidt S., Pirker S. (2019). Epidemiology and pathophysiology of autonomic seizures: A systematic review. Clin. Auton. Res..

[17] Fisher R.S., Cross J.H., French J.A., Higurashi N., Hirsch E., Jansen F.E., Lagae L., Moshé S.L., Peltola J., Roulet Perez E. (2017). Operational classification of seizure types by the International League Against Epilepsy: Position Paper of the ILAE Commission for Classification and Terminology. Epilepsia.

[18] Singh G., Sander J.W. (2020). The global burden of epilepsy report: Implications for low- and middle-income countries. Epilepsy Behav..

[19] Jovel C.A.E., Salazar S.R., Rodríguez C.R., Mejía F.E.S. (2016). Factors associated with quality of life in a low-income population with epilepsy. Epilepsy Res..

[20] Nazir N., Sabri A.A., Ahmad N., Akram M.N., Hussain H.A., Rasool A.G. (2020). Epidemiological study of epilepsy in Faisalabad. Prof. Med. J..

[21] Subota A., Khan S., Josephson C.B., Manji S., Lukmanji S., Roach P., Wiebe S., Buchhalter J., Federico P., Teskey G.C. (2019). Signs and symptoms of the postictal period in epilepsy: A systematic review and meta-analysis. Epilepsy Behav..

[22] DeGiorgio C.M., Curtis A., Carapetian A., Hovsepian D., Krishnadasan A., Markovic D. (2020). Why are epilepsy mortality rates rising in the United States? A population-based multiple cause-of-death study. BMJ Open.

[23] Mula M. (2018). Emerging drugs for focal epilepsy. Expert Opin. Emerg. Drugs.

[24] Quintana M., Sánchez-López J., Mazuela G., Santamarina E., Abraira L., Fonseca E., Seijo I., Álvarez-Sabin J., Toledo M. (2021). Incidence and mortality in adults with epilepsy in northern Spain. Acta Neurol. Scand..

[25] Nabbout R., Kuchenbuch M. (2020). Impact of predictive, preventive and precision medicine strategies in epilepsy. Nat. Rev. Neurol..

[26] Krasowski M.D., McMillin G.A. (2014). Advances in anti-epileptic drug testing. Clin. Chim. Acta.

[27] de Biase S., Nilo A., Bernardini A., Gigli G.L., Valente M., Merlino G. (2019). Timing use of novel anti-epileptic drugs: Is earlier better?. Expert Rev. Neurother..

[28] Mobed A., Shirafkan M., Charsouei S. (2022). Biosensors technology for anti-epileptic drugs. Clin. Chim. Acta.

[29] Málaga I., Sánchez-Carpintero R., Roldán S., Ramos-Lizana J., García-Peñas J.J. (2019). New anti-epileptic drugs in paediatrics. An. Pediatría (English Ed.).

[30] Rogawski M.A., Löscher W., Rho J.M. (2016). Mechanisms of Action of Antiseizure Drugs and the Ketogenic Diet. Cold Spring Harb. Perspect. Med..

[31] Peng S.J., Wong T.T., Huang C.C., Chang H., Hsieh K.L.C., Tsai M.L., Yang Y.S., Chen C.L. (2021). Quantitative analysis of intraoperative electrocorticography mirrors histopathology and seizure outcome after epileptic surgery in children. J. Formos. Med. Assoc..

[32] Consales A., Casciato S., Asioli S., Barba C., Caulo M., Colicchio G., Cossu M., de Palma L., Morano A., Vatti G. (2021). The surgical treatment of epilepsy. Neurol. Sci..

[33] Helmstaedter C., Beeres K., Elger C.E., Kuczaty S., Schramm J., Hoppe C. (2020). Cognitive outcome of pediatric epilepsy surgery across ages and different types of surgeries: A monocentric 1-year follow-up study in 306 patients of school age. Seizure.

[34] Numis A.L., da Gente G., Sherr E.H., Glass H.C. (2022). Whole-exome sequencing with targeted analysis and epilepsy after acute symptomatic neonatal seizures. Pediatr. Res..

[35] Janmohamed M., Brodie M.J., Kwan P. (2020). Pharmacoresistance—Epidemiology, mechanisms, and impact on epilepsy treatment. Neuropharmacology.

[36] Zarnowska I.M. (2020). Therapeutic Use of the Ketogenic Diet in Refractory Epilepsy: What We Know and What Still Needs to Be Learned. Nutrients.

[37] Tekin E., Serdaroğlu F.M., Şahin Ş., Taşdemir H.A. (2021). Ketogenic diet experience at Ondokuz Mayıs University. Neurol. Sci..

[38] Li R.J., Liu Y., Liu H.Q., Li J. (2020). Ketogenic diets and protective mechanisms in epilepsy, metabolic disorders, cancer, neuronal loss, and muscle and nerve degeneration. J. Food Biochem..

[39] Dabek A., Wojtala M., Pirola L., Balcerczyk A. (2020). Modulation of Cellular Biochemistry, Epigenetics and Metabolomics by Ketone Bodies. Implications of the Ketogenic Diet in the Physiology of the Organism and Pathological States. Nutrients.

[40] Wells J., Swaminathan A., Paseka J., Hanson C. (2020). Efficacy and Safety of a Ketogenic Diet in Children and Adolescents with Refractory Epilepsy—A Review. Nutrients.

[41] Dhillon K.K., Gupta S. (2022). Biochemistry, Ketogenesis; StatPearls Publishers; StatPearls [Internet]. https://www.ncbi.nlm.nih.gov/pubmed/29630231.

[42] Masino S.A., Li T., Theofilas P., Sandau U.S., Ruskin D.N., Fredholm B.B., Geiger J.D., Aronica E., Boison D. (2011). A ketogenic diet suppresses seizures in mice through adenosine A1 receptors. J. Clin. Investig..

[43] Yang H., Shan W., Zhu F., Wu J., Wang Q. (2019). Ketone Bodies in Neurological Diseases: Focus on Neuroprotection and Underlying Mechanisms. Front. Neurol..

[44] Rosenbaum M., Hall K.D., Guo J., Ravussin E., Mayer L.S., Reitman M.L., Smith S.R., Walsh B.T., Leibel R.L. (2019). Glucose and Lipid Homeostasis and Inflammation in Humans Following an Isocaloric Ketogenic Diet. Obesity.

[45] Dunwiddie T.V., Diao L., Proctor W.R. (1997). Adenine Nucleotides Undergo Rapid, Quantitative Conversion to Adenosine in the Extracellular Space in Rat Hippocampus. J. Neurosci..

[46] Kawamura M., Ruskin D.N., Masino S.A. (2010). Metabolic Autocrine Regulation of Neurons Involves Cooperation among Pannexin Hemichannels, Adenosine Receptors, and KATP Channels. J. Neurosci..

[47] Mao X.-Y., Zhou H.-H., Jin W.-L. (2019). Redox-Related Neuronal Death and Crosstalk as Drug Targets: Focus on Epilepsy. Front. Neurosci..

[48] Plum L. (2006). Enhanced PIP3 signaling in POMC neurons causes KATP channel activation and leads to diet-sensitive obesity. J. Clin. Investig..

[49] Singh B., Khattab F., Chae H., Desmet L., Herrera P.L., Gilon P. (2021). KATP channel blockers control glucagon secretion by distinct mechanisms: A direct stimulation of α-cells involving a [Ca^2+^]_c_ rise and an indirect inhibition mediated by somatostatin. Mol. Metab..

[50] Yang H.Q., Echeverry F.A., ElSheikh A., Gando I., Arredondo S.A., Samper N., Cardozo T., Delmar M., Shyng S.L., Coetzee W.A. (2022). Subcellular trafficking and endocytic recycling of K ATP channels. Am. J. Physiol. Physiol..

[51] Lutas A., Yellen G. (2013). The ketogenic diet: Metabolic influences on brain excitability and epilepsy. Trends Neurosci..

[52] Ułamek-Kozioł M., Czuczwar S.J., Januszewski S., Pluta R. (2019). Ketogenic Diet and Epilepsy. Nutrients.

[53] Kayode O.T., Rotimi D.E., Afolayan O.A., Kayode A.A. (2020). Ketogenic diet: A nutritional remedy for some metabolic disorders. J. Educ. Health Sport..

[54] Hartman A.L., Gasior M., Vining E.P., Rogawski M.A. (2007). The Neuropharmacology of the Ketogenic Diet. Pediatr. Neurol..

[55] Murakami M., Tognini P. (2022). Molecular Mechanisms Underlying the Bioactive Properties of a Ketogenic Diet. Nutrients.

[56] Molteberg E., Thorsby P.M., Kverneland M., Iversen P.O., Selmer K.K., Nakken K.O., Taubøll E. (2020). Effects of modified Atkins diet on thyroid function in adult patients with pharmacoresistant epilepsy. Epilepsy Behav..

[57] Cervenka M.C., Barron B.J., Kossoff E.H., Zahava Turner R.D. (2016). The Ketogenic and Modified Atkins Diets: Treatments for Epilepsy and Other Disorders. https://ebookcentral.proquest.com/lib/unc/detail.action?docID=4429634.

[58] Dou X., Xu X., Mo T., Chen H., Wang Z., Li X., Jia S., Wang D. (2022). Evaluation of the seizure control and the tolerability of ketogenic diet in Chinese children with structural drug-resistant epilepsy. Seizure.

[59] Kossoff E.H., Zupec-Kania B.A., Amark P.E., Ballaban-Gil K.R., Christina Bergqvist A.G., Blackford R., Buchhalter J.R., Caraballo R.H., Helen Cross J., Dahlin M.G. (2009). Optimal clinical management of children receiving the ketogenic diet: Recommendations of the International Ketogenic Diet Study Group. Epilepsia.

[60] Feinman R.D. (2020). The biochemistry of low-carbohydrate and ketogenic diets. Curr. Opin. Endocrinol. Diabetes Obes..

[61] Longo R., Peri C., Cricrì D., Coppi L., Caruso D., Mitro N., De Fabiani E., Crestani M. (2019). Ketogenic Diet: A New Light Shining on Old but Gold Biochemistry. Nutrients.

[62] Bruci A., Tuccinardi D., Tozzi R., Balena A., Santucci S., Frontani R., Mariani S., Basciani S., Spera G., Gnessi L. (2020). Very Low-Calorie Ketogenic Diet: A Safe and Effective Tool for Weight Loss in Patients with Obesity and Mild Kidney Failure. Nutrients.

[63] Stubbs B.J., Koutnik A.P., Goldberg E.L., Upadhyay V., Turnbaugh P.J., Verdin E., Newman J.C. (2020). Investigating Ketone Bodies as Immunometabolic Countermeasures against Respiratory Viral Infections. Med.

[64] Morris G., Puri B.K., Carvalho A., Maes M., Berk M., Ruusunen A., Olive L. (2020). Induced Ketosis as a Treatment for Neuroprogressive Disorders: Food for Thought?. Int. J. Neuropsychopharmacol..

[65] Madaan P., Jauhari P., Chakrabarty B., Gulati S. (2019). Ketogenic Diet in Epilepsy of Infancy with Migrating Focal Seizures. Pediatr. Neurol..

[66] Green S.F., Nguyen P., Kaalund-Hansen K., Rajakulendran S., Murphy E. (2020). Effectiveness, retention, and safety of modified ketogenic diet in adults with epilepsy at a tertiary-care centre in the UK. J. Neurol..

[67] Tian X., Chen J., Zhang J., Yang X., Ji T., Zhang Y., Wu Y., Fang F., Wu X., Zhang Y. (2019). The Efficacy of Ketogenic Diet in 60 Chinese Patients with Dravet Syndrome. Front Neurol..

[68] Lucchi C., Marchiò M., Caramaschi E., Giordano C., Giordano R., Guerra A., Biagini G. (2017). Electrographic Changes Accompanying Recurrent Seizures under Ketogenic Diet Treatment. Pharmaceuticals.

[69] Marchiò M., Roli L., Giordano C., Trenti T., Guerra A., Biagini G. (2019). Decreased ghrelin and des-acyl ghrelin plasma levels in patients affected by pharmacoresistant epilepsy and maintained on the ketogenic diet. Clin. Nutr..

[70] Masino S.A., Rho J.M. (2019). Metabolism and epilepsy: Ketogenic diets as a homeostatic link. Brain Res..

[71] Grigolon R.B., Gerchman F., Schöffel A.C., Hawken E.R., Gill H., Vazquez G.H., Mansur R.B., McIntyre R.S., Brietzke E. (2020). Mental, emotional, and behavioral effects of ketogenic diet for non-epileptic neuropsychiatric conditions. Prog. Neuro Psychopharmacol. Biol. Psychiatry.

[72] Boison D., Steinhäuser C. (2018). Epilepsy and astrocyte energy metabolism. Glia.

[73] Simeone T.A., Simeone K.A., Stafstrom C.E., Rho J.M. (2018). Do ketone bodies mediate the anti-seizure effects of the ketogenic diet?. Neuropharmacology.

[74] Barzegar M., Afghan M., Tarmahi V., Behtari M., Rahimi Khamaneh S., Raeisi S. (2021). Ketogenic diet: Overview, types, and possible anti-seizure mechanisms. Nutr. Neurosci..

[75] Olson C.A., Vuong H.E., Yano J.M., Liang Q.Y., Nusbaum D.J., Hsiao E.Y. (2018). The Gut Microbiota Mediates the Anti-Seizure Effects of the Ketogenic Diet. Cell.

[76] Schwantje M., Verhagen L.M., van Hasselt P.M., Fuchs S.A. (2020). Glucose transporter type 1 deficiency syndrome and the ketogenic diet. J. Inherit. Metab. Dis..

[77] Ali A.M., Kunugi H. (2020). Intermittent Fasting, Dietary Modifications, and Exercise for the Control of Gestational Diabetes and Maternal Mood Dysregulation: A Review and a Case Report. Int. J. Environ. Res. Public Health.

[78] Zhu T.W., Zhang X., Zong M.H., Linhardt R.J., Wu H., Li B. (2020). Storage stability studies on interesterified blend-based fast-frozen special fats for oxidative stability, crystallization characteristics and physical properties. Food Chem..

[79] Manville R.W., Abbott G.W. (2020). Potassium channels act as chemosensors for solute transporters. Commun. Biol..

[80] El-Rashidy O.F., Youssef M.M., Elgendy Y.G., Mohsen M.A., Morsy S.M., Dawh S.A., Saad K. (2020). Selenium and antioxidant levels in children with intractable epilepsy receiving ketogenic diet. Acta Neurol. Belg..

[81] Mastrangelo M., Tromba V., Silvestri F., Costantino F. (2019). Epilepsy in children with type 1 diabetes mellitus: Pathophysiological basis and clinical hallmarks. Eur. J. Paediatr. Neurol..

[82] Khodabakhshi A., Akbari M.E., Mirzaei H.R., Mehrad-Majd H., Kalamian M., Davoodi S.H. (2020). Feasibility, Safety, and Beneficial Effects of MCT-Based Ketogenic Diet for Breast Cancer Treatment: A Randomized Controlled Trial Study. Nutr. Cancer.

[83] Goswami J.N., Sharma S. (2019). Current Perspectives On The Role Of The Ketogenic Diet In Epilepsy Management. Neuropsychiatr. Dis. Treat..

[84] Brietzke E., Mansur R.B., Subramaniapillai M., Balanzá-Martínez V., Vinberg M., González-Pinto A., Rosenblat J.D., Ho R., McIntyre R.S. (2018). Ketogenic diet as a metabolic therapy for mood disorders: Evidence and developments. Neurosci. Biobehav. Rev..

[85] McDonald T.J., Henry-Barron B.J., Felton E.A., Gutierrez E.G., Barnett J., Fisher R., Lwin M., Jan A., Vizthum D., Kossoff E.H. (2018). Improving compliance in adults with epilepsy on a modified Atkins diet: A randomized trial. Seizure.

[86] Di Lorenzo C., Coppola G., Di Lenola D., Evangelista M., Sirianni G., Rossi P., Di Lorenzo G., Serrao M., Pierelli F. (2018). Efficacy of Modified Atkins Ketogenic Diet in Chronic Cluster Headache: An Open-Label, Single-Arm, Clinical Trial. Front. Neurol..

[87] Ludwig D.S. (2020). The Ketogenic Diet: Evidence for Optimism but High-Quality Research Needed. J. Nutr..

[88] Khattak S.H., Begum S., Aqeel M., Fayyaz M., Bangash S.A.K., Riaz M.N., Saeed S., Ahmed A., Ali G.M. (2020). Investigating the Allelic Variation of Loci Controlling Rust Resistance Genes in Wheat (Triticum aestivum L.) Land Races by Ssr Marker. Appl. Ecol. Environ. Res..

[89] Qaiser R., Fayyaz M., Sufiyan M., Khattak S.H., Sandhu K.S., Sidhu G.S. (2020). Genome-wide association mapping and population structure for stripe rust in Pakistani wheat germplasm. Pakistan J. Bot..

[90] Waqar A., Khattak S.H., Begum S., Rehman T., Shehzad A., Ajmal W., Zia S.S., Siddiqi I., Ali G.M. (2018). Stripe Rust: A Review of the Disease, Yr Genes and its Molecular Markers. Sarhad J. Agric..

[91] Begum S., Khan M.R., Jan A., Rahman H., Khattak S.H., Saeed S., Ahmed A., Ali G.M. (2022). Genetic Transformation of the Epsps Herbicide Resistance Gene in Agrobacterium Mediated Peanut (*Arachis hypogaea* L.) and Effective Revival of Transgenic Plants. Appl. Ecol. Environ. Res..

[92] Zupec-Kania B. (2008). KetoCalculator: A web-based calculator for the ketogenic diet. Epilepsia.

[93] Seo J.G., Cho Y.W., Kim K.T., Kim D.W., Yang K.I., Lee S.T., Byun J.I., No Y.J., Kang K.W., Kim D. (2020). Pharmacological Treatment of Epilepsy in Elderly Patients. J. Clin. Neurol..

[94] van der Louw E., van den Hurk D., Neal E., Leiendecker B., Fitzsimmon G., Dority L., Thompson L., Marchió M., Dudzińska M., Dressler A. (2016). Ketogenic diet guidelines for infants with refractory epilepsy. Eur. J. Paediatr. Neurol..

[95] Park E.G., Lee J., Lee J. (2018). Use of the Modified Atkins Diet in Intractable Pediatric Epilepsy. J. Epilepsy Res..

[96] Armeno M., Araujo C., Sotomontesano B., Caraballo R.H. (2018). Update on the adverse effects during therapy with a ketogenic diet in paediatric refractory epilepsy. Rev. Neurol..

[97] Heussinger N., Della Marina A., Beyerlein A., Leiendecker B., Hermann-Alves S., Dalla Pozza R., Klepper J. (2018). 10 patients, 10 years—Long term follow-up of cardiovascular risk factors in Glut1 deficiency treated with ketogenic diet therapies: A prospective, multicenter case series. Clin. Nutr..

[98] Kapoor D., Garg D., Sharma S. (2021). Emerging role of the ketogenic dietary therapies beyond epilepsy in child neurology. Ann. Indian Acad. Neurol..

[99] Mosek A., Natour H., Neufeld M.Y., Shiff Y., Vaisman N. (2009). Ketogenic diet treatment in adults with refractory epilepsy: A prospective pilot study. Seizure.

[100] Kossoff E.H., Al-Macki N., Cervenka M.C., Kim H.D., Liao J., Megaw K., Nathan J.K., Raimann X., Rivera R., Wiemer-Kruel A. (2015). What are the minimum requirements for ketogenic diet services in resource-limited regions? Recommendations from the International League Against Epilepsy Task Force for Dietary Therapy. Epilepsia.

